# A potent peptide as adiponectin receptor 1 agonist to against fibrosis[Author-notes FN0001]

**DOI:** 10.1080/14756366.2017.1284067

**Published:** 2017-03-06

**Authors:** Lingman Ma, Zhen Zhang, Xiaowen Xue, Yumeng Wan, Boping Ye, Kejiang Lin

**Affiliations:** a Department of Medicinal Chemistry, School of Pharmacy, China Pharmaceutical University, Nanjing, China;; b School of Life Science and Technology, China Pharmaceutical University, Nanjing, China;; c Department of Pharmacy, First People’s Hospital of Changde City, Changde, Hunan, China

**Keywords:** adiponectin receptor 1, homology modelling, molecular docking, virtual screening, anti-fibrotic activity

## Abstract

Fibrotic diseases have become a major cause of death in the developed world. AdipoR1 agonists are potent inhibitors of fibrotic responses. Here, we focused on the *in silico* identification of novel AdipoR1 peptide agonists. A homology model was constructed to predict the 3D structure of AdipoR1. By docking to known active peptides, the putative active site of the model was further explored. A virtual screening study was then carried out with a set of manually designed peptides using molecular docking. Peptides with high docking scores were then evaluated for their anti-fibrotic properties. The data indicated that the novel peptide Pep70 significantly inhibited the proliferation of hepatic stellate cells (HSC) and NIH-3T3 cells (18.33% and 27.80%) and resulted in favouring cell-cycle arrest through increasing the accumulation of cells in the G0/G1 phase by 17.08% and 15.86%, thereby reducing the cell population in the G2/M phase by 11.25% and 15.95%, respectively. Additionally, Pep70 exhibited the most marked suppression on the expression of α-smooth muscle actin (α-SMA), collagen type I alpha1 (COL1A1) and TGF-β1. Therefore, the peptide Pep70 was ultimately identified as an inhibitor of fibrotic responses and as a potential AdipoR1 agonist.

## Introduction

Fibrosis is defined by overgrowth, hardening, and/or scarring of various tissues due to the excessive accumulation of extracellular matrix (ECM) components such as collagen and fibronectin^1^^,^^2^. Different fibrotic diseases have different aetiologies, but they all share a common pathogenic process: excessive activation of TGF-β that initiates and sustains fibroblast activation and myofibroblast differentiation^3^. Thus, blocking the TGF-β signal transduction pathway has been explored for the treatment of fibrotic diseases^4^. Recent studies demonstrate that activated adiponectin receptor can block the TGF-β pathway via AMPK, which can inhibit the secretion of extracellular matrix protein collagen type I (COL1A1) and prevent the induction of the myofibroblast phenotype marker α-SMA^5^^,^^6^. Adiponectin, a 244-amino acid protein secreted predominantly by white adipose tissue^7^, activates the AMPK pathway through interacting with AdipoR1, which is a subtype of adiponectin receptor, to offer protection against hepatic fibrogenesis^8^. Due to its larger peptide fragments, the clinical applications of complete adiponectin protein are restricted. Therefore, the development of simpler and smaller molecules associated with adiponectin that could function as adiponectin receptor agonist became a focus of some researchers. Laszlo Otvos Jr^9^ found six adiponectin-based peptide compounds acting as AdipoR1 agonists through screening a panel of 66 overlapping peptides, each 10 amino acids long, covering the entire globular domain of the human adiponectin protein. One of these peptides, named ADP355, mimics key biological functions of adiponectin *in vitro* as well as *in vivo*. Marco Miele^10^ found a nine-residue osmotin peptide that can interact with AdipoR1 via studying the structural and functional similarities between osmotin and human adiponectin.

In this study, we created a homology model of AdipoR1 *in silico* with a seven-transmembrane domain, of which the N-terminus is internal and the C-terminus is external, in contrast to the topology of all reported G protein-coupled receptors (GPCRs)^11^. The putative active site of the model was further explored by docking with the known active peptides^9^^,^^10^. A virtual screening study was carried out with a set of manually designed peptides using molecular docking. Furthermore, peptides with high docking scores were evaluated for their anti-fibrotic bioactivity levels, and one new peptide with promising anti-fibrotic effects was subjected to a binding mode analysis.

## Materials and methods

### Materials

Pep56, Pep69, Pep70, Pep71 and ADP355 (purity ≥98%) was synthesised using standard Fmoc solid-phase synthesis protocols (Shanghai Top-peptide Biotechnology Co., Ltd, Shanghai, China); CCK-8 assay kit (Dojindo Molecular Technologies Inc., Rockville, MD); Rat TGF-β1 protein (Beijing Creanove Biotechnolpgy Co., Ltd, Beijing, China); α-SMA Rb pAB, COL1A1 Rb pAB and TGF-β1 Rb pAB (Wanleibio, Shenyang, China); rabbit β-actin antibody (CST, Danvers, MA); BCA protein assay kit (Beyotime Biotechnology, Shanghai, China); Trizol reagent (Invitrogen, Carlsbad, CA); HiFiScript 1st Strand cDNA Synthesis Kit and UltraSYBR Mixture (ComWin Biotech Co., Ltd, Jiangsu, China).

### Methods

#### Modelling of AdipoR1

The amino acid sequence of AdipoR1 (NP_057083.2) was retrieved from the NCBI database. The template structure (PDB ID: 1GU8) was obtained from the PDB based on the report of Tanabe et al.^10^. The Build Homology Models module in DS was applied for homology modelling. The number of models was set to 10, and the optimisation level was changed to high. Refinement loops were set to true, and the other parameters remained as their defaults. The best model among the model structures generated was chosen on the basis of the PDF total energy and DOPE score, the two basic functions in DS for model evaluation. The chosen model was subjected to energy minimisation and molecular dynamics simulation. Given that AdipoR1 belongs to the transmembrane protein family, a membrane was added using the Create and Edit Membrane module in DS before energy minimisation. Generalized Born with Implicit Membrane (GBIM) was selected as the implicit solvent model. The system was refined using the CHARMm forcefield with the Smart Minimiser protocol (Steepest Descent method followed by Conjugate Gradient method). The Standard Dynamics Cascade module was applied for molecular dynamics simulation. Other parameters remained at their defaults except for the use of GBIM as the implicit solvent model. A Ramachandran plot and the Profile-3D module in DS were used to evaluate the optimised model.

#### Virtual screening

Virtual screening based on molecular docking is an effective method for the identification of novel compounds. The programme GOLD 5.1 was employed for our docking virtual screen. Genetic Optimisation for Ligand Docking (GOLD) is a genetic algorithm for docking compounds into protein binding sites. Considering full ligand flexibility and partial protein flexibility including protein side chains and backbone flexibility), GOLD shows high accuracy and reliability^12^^,^^13^. Docking studies of the known active peptide was first applied to explore the probable active site of the protein model. A set of manually designed peptides was generated using the Build and Edit Protein module in DS for virtual screening. The construction of the peptides started with the known active peptide, and we changed residues based on the rules provided by Laszlo Otvos Jr^9^. A goldscore fitness function was used to evaluate the docking results, and peptides with high goldscores were selected for *in vitro* experiments.

#### The anti-fibrotic experiment

##### Cell culture

The rat immortalised HSC line HSC-T6 and mouse NIH-3T3 cells were cultured at 37 °C with 5% CO_2_ in Dulbecco’s modified Eagle’s medium (DMEM) with a high glucose concentration (4.5 g/L) supplemented with 10% foetal bovine serum (FBS) and 1% penicillin/streptomycin.

##### Cell proliferation assay

The cell viability of HSC-T6 and NIH-3T3 cells was determined by CCK-8 assay according to the instructions of the manufacturer. Briefly, cells were plated onto 96-well plates (1 × 10^4^ cells/well) and treated with different concentrations of peptides (0.05, 0.5, 1.0, 5.0, 10.0, 50 and 100 μM) or PBS for 24 h. CCK-8 solutions were added and incubated for 2 h followed by viable cell detection with a microplate reader at 450 nm absorbance (Model 550; Bio-Rad, Hercules, CA).

##### Flow cytometric analysis of the cell cycle

For cell-cycle analysis, cells were incubated in a 24-well plate (1 × 10^4^ cells/well) and pre-treated with peptides (10, 50 and 100 μM) or PBS for 1 h followed by 24 h co-incubation with rat TGF-β1 (5 ng/ml). Cells were collected and fixed in cold 70% ethanol overnight at 4 °C, followed by PI (1 mg/ml) staining in the presence of 1% RNase A for 30 min at 37 °C in the dark. The percentages of cells in the sub-G1, G0/G1, S and G2/M phases were analysed by flow-cytometry (BD FACSCalibur, San Jose, BD) at an excitation and emission of 488 nm and 630 nm, respectively. At least 15,000 cells were harvested for each analysis, and the experiments were performed in triplicate.

##### Western blot analysis

After 16 h of synchronised growth in serum-free media containing 0.1% FBS, HSC-T6 and NIH-3T3 cells were pre-treated with 10 μM peptides or PBS for 1 h, followed by 24 h co-incubation with rat TGF-β1 (5 ng/ml), washed with ice-cold HBSS, and then collected by gentle scraping. The total protein was quantified using the bicinchoninic acid (BCA) protein assay kit. A total of 30 μg of each protein lysate (RIPA lysis buffer) was mixed with 5 × Laemmli sample buffer and subjected to 10% SDS-PAGE. Blots were probed with α-SMA, COL1A1 and TGF-β1 antibody.

##### Real-time qPCR

To investigate the screened peptides effects on the expression of fibrotic genes (*α-SMA*, *COL1A1* and *TGF-β1*), HSC-T6 and NIH-3T3 cells were treated with different concentrations of peptides or PBS for 1 h, followed by 24 h co-incubation with rat TGF-β1 (final concentration 5 ng/ml). After total RNA extraction, reverse transcription was performed using 400 ng RNA according to the instructions of the manufacturer. The primers for qPCR are listed in Table 1. Real-time PCR was carried out using a CFX96 Optical Reaction Module for Real-Time PCR Systems (Bio-Rad Laboratories Inc., Hercules, CA). The expression levels of three fibrotic genes were normalised against β-actin and compared with the model group (only rat TGF-β1 stimulation). Data were showed as the mean of two experiments, and three parallel samples were prepared for each peptide concentration.

**Table 1.** Primers used for real-time qPCR.

Gene		Primer sequences	NCBI number
Rat α-SMA	F	5′-GATCACCATCGGGAATGAACGC-3′	NM_031004
	R	5′-CTTAGAAGCATTTGCGGTGGAC-3′	
Rat COL1A1	F	5′-TGCCGTGACCTCAAGATGTG-3′	NM_053304.1
	R	5′-CACAAGCGTGCTGTAGGTGA-3′	
Rat TGF-β1	F	5′-TGGCGT TACCTTGGTAACC-3′	NM_021578
	R	5′-GGT GTT GAGCCCTTTCCAG-3′	
Rat 18S	F	5′-GTAACCCGTTGAACCCCATT-3′	100861533
	R	5′-CCATCCAATCGGTAGTAG CG-3′	

α-SMA: α-smooth muscle actin; COL1A1: collagen type I alpha1; TGF-β1: transforming growth factor-β1.

## Results and discussion

### Modelling of AdipoR1

The homology model of AdipoR1 was built using the Discovery Studio (DS) software (Accelrys, San Diego, CA). This programme can simultaneously incorporate structural data from one or more reference proteins. Structural features in the reference proteins are used to derive spatial restraints, which in turn are used to generate model protein structures using conjugate gradients and simulated annealing optimisation procedures. Building on the strategy of Marco Miele^10^, protein rhodopsin II (PDB code: 1GU8) was used as the reference protein. The probability density function (PDF) total energy and Discrete Optimised Protein Energy (DOPE) scores were used for model evaluation. The PDF total energy is the sum of the scoring function value of all homology-derived pseudo-energy terms and stereochemical pseudo-energy terms, while the DOPE score of a protein is a conformational energy that measures the relative stability of a conformation with respect to other conformations of the same protein^14^. Both can assist in choosing the best model out of a set of predicted model structures of a protein sequence. In total, 20 models were obtained (Table 2) and based on its lowest PDF total energy and relatively low DOPE scores. The best model was selected for further optimisation by energy minimisation and molecular dynamics simulation.

**Table 2.** PDF total energy and DOPE scores of each model.

Name	PDF total energy	PDF physical energy	DOPE scores
Model 1	−6101.6001	−6223.795288	−29,642.783203
Model 2	−5951.4771	−6045.5263	−29,085.187500
Model 3	−5944.6123	−6053.62447	−29,895.927734
Model 4	−5825.3857	−6077.285249	−29,780.341797
Model 5	−5588.0986	−5909.8098	−29,336.101563
Model 6	−4744.4258	−5377.0285	−28,973.908203
Model 7	−4453.9067	−5147.75016	−28,890.529297
Model 8	−3857.1067	−4976.139369	−29,153.625000
Model 9	−1251.0862	−4051.5576	−29,059.587891
Model 10	4628.9204	−3713.2433	−28,837.755859
Model 11	838.0331	520.8693674	−29,528.294922
Model 12	856.4171	534.0826223	−30,152.189453
Model 13	907.9245	540.0810716	−30,236.638672
Model 14	911.5750	536.2312286	−29,739.263672
Model 15	960.4460	570.715712	−29,998.464844
Model 16	1029.5458	591.6176848	−29,243.019531
Model 17	1030.5454	547.0577104	−29,656.089844
Model 18	1040.6725	568.3925966	−29,448.673828
Model 19	1042.4070	575.00610873	−29,382.707031
Model 20	1063.9772	569.31939989	−30,063.341797

**Figure 1.**
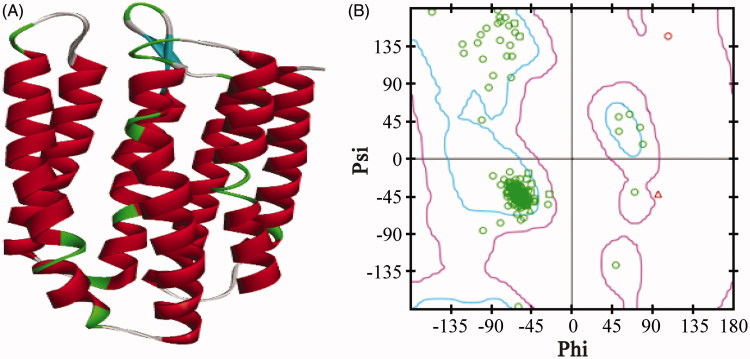
The 3D structure of the AdipoR1 homology model (A) and the Ramachandran plot of model 1 (B). The zone in the blue line (inside line) was the most favoured region, and the zone in the purple line (outside line) was the allowed region. The zone outside of the purple line was the disallowed region.

The reliability of the model was assessed and confirmed by comparing specific model scores with the “expected high” and “expected low” scores generated by the Verify Protein (Profiles-3D) module in the DS programme. The optimised model 1 generated a score of 97.63, which was closer to the expected high score of 112.68 than to the expected low score of 50.71. This result confirmed that the optimised model 1 was reliable (Figure 1A). A Ramachandran plot provides a graphical representation of the local backbone conformation of each residue in a protein and includes a representation of the favourable and unfavourable regions for residues^15^. As shown in Figure 1(B), only two residues in the generated structure fell in the unfavourable region, while 93.8% residues fell in the most favoured region, which further validated the quality of model 1.

### The mapping of active binding sites by docking with known active peptides

In Laszlo Otvos Jr’s work^9^, they examined the interaction of an active peptide with biotin-labelled fragments of the 4 AdipoR1 extracellular loops. They found that the active peptide bound the first extracellular loop of AdipoR1. Therefore, we defined the residues of loop 1 as the binding site; the coordinates of the binding site sphere were *x* = 5.2647, *y* = −3.849 and *z* =  −25.191, and the radius was 15 Å. The GOLD programme was applied to dock the optimised model 1 to the known active peptides (the sequence of each peptide is listed in Table 3). Docking with each peptide generated 10 docking poses, and the poses with highest goldscores were selected to achieve complex system balance by molecular dynamics simulation. The final poses were chosen for the analysis of the interaction between the peptides and model 1. The residues involved in the interaction of each peptide are listed in Table 3. K35 was identified as a key residue because it was involved in all of the peptide-protein interactions except for Pep23. To make the virtual screening more accurate, we redefined the binding site with residue K35 set as the centre of the sphere, and we reset the coordinates of the binding site sphere (*x*, *y*, *z*: 5.913, −4.498 and −24.006, and radius 9 Å).

**Table 3.** Sequence of known active peptides and the docking results to the model 1.

Name	Sequence	Goldscores	Interacting residues in AdipoR1
Peposm	CTQGPCGPT	66.79	K35, V220
ADP355	NIPXLYSFAS	92.77	P24, A30, E34, K35, G219
Pep23	KFHCNIPGLY	93.46	H216
Pep24	HCNIPGLYYF	92.66	Q33, K35
Pep25	NIPGLYYFAY	72.77	K35, M21, Y218, F156
Pep26	PGLYYFAYHI	90.05	K35, F38, S92, Q164, H216
Pep27	LYYFAYHITV	98.75	K35

### Identifying biologically active compounds through virtual screening

In silico virtual high-throughput screening is a rapid method of identifying the biologically active compounds. A virtual screening based on docking was used to identify the peptides that can effectively interact with optimised model 1. A total of 747 manually designed peptides were included in the screen library. The top three peptides in goldscores were selected for further anti-fibrotic bioactivity studies (Table 4).

**Table 4.** Sequence and gold scores of the screened peptides.

Name	Sequence	Goldscores
Pep69	KFYIHSDY	101.55
Pep70	PGLYYFD	108.01
Pep71	FSYHSF	98.60

### The anti-fibrotic properties of the screened peptides

#### Pep70 inhibited the cell proliferation of HSC-T6 and NIH-3T3

**Figure 2.**
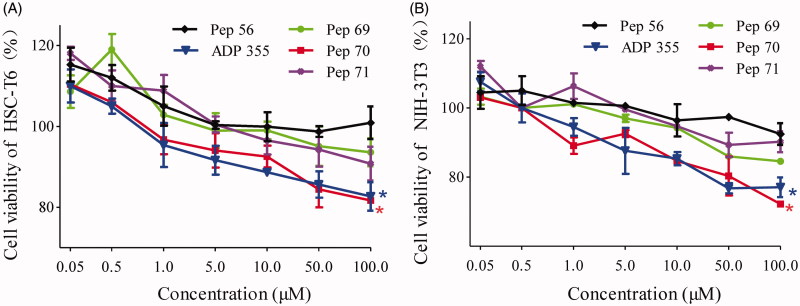
The cell proliferation of HSC-T6 and NIH-3T3 cells treated with different concentrations of peptide or PBS. (A) Cell proliferation of HSC-T6 cells. (B) Cell proliferation of NIH-3T3 cells. Statistically different levels are denoted by *p < 0.01, and the data are presented as the mean of three repeated experiments.

The activation of HSCs is the crucial step of fibrogenesis because HSCs are the main producers responsible for the excessive production of extracellular matrix and profibrogenic cytokines in fibrotic livers^16^^,^^17^. Obtained from desegregated NIH Swiss mouse embryo fibroblasts, NIH-3T3 cells have become a standard fibroblast cell line^18^^,^^19^. To investigate the anti-proliferative effect of the studied peptides, HSC-T6 and NIH-3T3 cells were treated with different concentrations of peptides as indicated, and then the cell viability was determined by CCK-8 assay. ADP355^20–22^, a novel adiponectin-based short peptide agonist (H-DAsn-Ile-Pro-Nva-Leu-Tyr-DSer-Phe-Ala-DSer-NH2) with potent anti-fibrotic activities *in vivo* and *in vitro*, was used as a positive control. Pep56, a sequence-scrambled peptide, was used as a negative control. As Figure 2 shows, cell proliferation for HSC-T6 and NIH-3T3 cells was significantly inhibited by Pep70. At a dose of 10 μM, the suppression rates against NIH-3T3 cells reached 15.3%. Notably, this anti-proliferation effect of Pep70 was similar to ADP355, while Pep69 and Pep71 exhibited lower suppression rates compared with Pep70. Several kinds of peptides harbour the capacity to prevent fibrosis driven by HSC-T6 cells^23^^,^^24^, and Pep70 also attenuates fibrosis by suppressing the proliferation of HSC-T6 and NIH-3T3 cells.

#### Pep70-induced cell-cycle arrest

**Figure 3.**
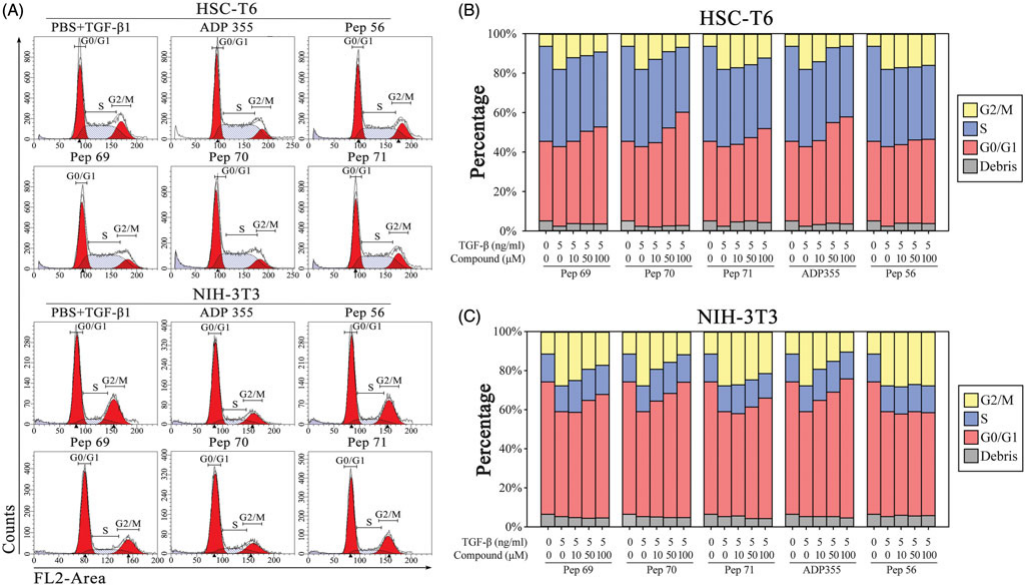
Pep70-induced cell-cycle arrest. Flow cytometric cell-cycle analysis in HSC-T6 and NIH-3T3 cells subjected to different concentrations of peptide treatment under 5 ng/ml rat TGF-β1 stimulation for 24 h. (A) Flow cytometric histograms showing cell distributions in the G0/G1, S and G2/M phases of the cell cycle. (B and C) Quantification of the DNA content in the G0/G1, S and G2/M phases. This study was conducted in duplicate, and the data from one representative experiment are shown.

Restrained proliferation always presents as cell-cycle arrest^25^. To further examine the anti-proliferative effect of the screened peptides, HSC-T6 and NIH-3T3 cells were used to determine the cell-cycle perturbations using flow cytometry. In untreated NIH-3T3 cells, the cell-cycle distribution curve showed 53.64% of the population in the G0/G1 phase, 27.79% of the population in the G2/M phase and 18.57% of the population in the S phase, explaining the rapid proliferation of the cells. Upon treatment with Pep70, the cells were arrested in the G0/G1 phase with 69.50% of the population in the G0/G1 phase and 11.84% of the population in the G2/M phase, with no obvious increase in the apoptotic sub-G1 fraction (Figure 3C). For HSC-T6 cells, Pep70 treatment also favoured cell-cycle arrest by increasing the cells’ accumulation in the G0/G1 phase by 17.08% and reducing the cell population in the G2/M phase by 11.25% compared with untreated cells, which stayed in the same phase (57.50% versus 40.42% and 6.84% versus 18.09%, respectively), as shown in Figure 3(B). The cell-cycle perturbations of Pep70 were similar to that of ADP355, suggesting its obvious anti-fibrotic effects.

#### Pep70 suppressed TGF-β-induced gene and protein expression of α-SMA, COL1A1 and TGF-β1

**Figure 4.**
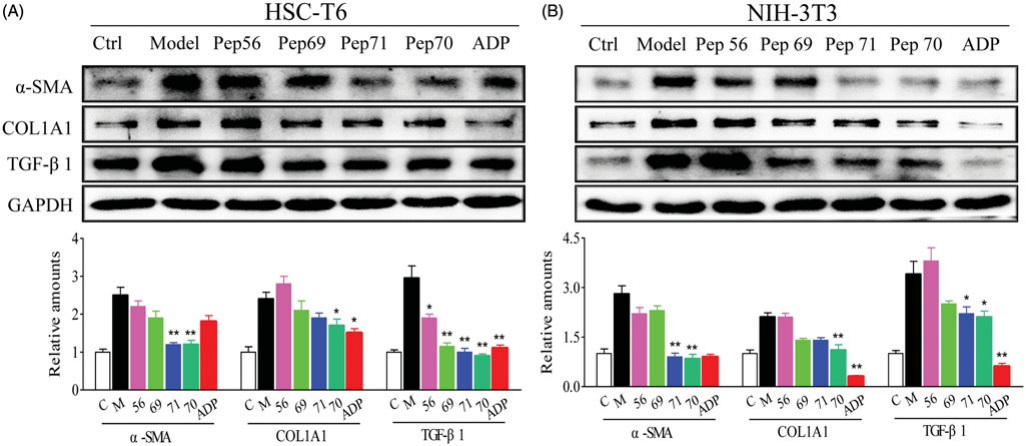
Representative western blot analysis of HSC-T6 (A) and NIH-3T3 (B) cells treated with PBS or 10 μM screened peptides in the presence of 5 ng/ml rat TGF-β1 for 24 h. The protein expression levels of α-SMA, COL1A1 and TGF-β1 were probed. GAPDH served as the loading control, and PBS served as the control (Ctl), while only 5 ng/ml TGF-β1 stimulation served as the model (Model). This experiment was repeated three times under the same conditions, and one representative set of results are presented.

A hallmark of HSC activation is overexpression of α-SMA and increased production of COL1A1 and fibronectin^26^^,^^27^. Additionally, the activation of HSCs increases the responsiveness to TGF-β1 through the up-regulation or *de novo* expression of TGF-β1 receptors^28^. Therefore, TGF-β1 is an established and potent profibrogenic mediator in the progression of liver fibrosis^29^^,^^30^. Here, rat TGF-β1 was used as a fibrosis stimulant in cultured fibroblasts. Blocking the TGF-β signalling pathway can inhibit the fibrotic response by leading to the down-regulated expression of COL1A1 induced by TGF-β and the myofibroblast phenotype marker α-SMA. To investigate the anti-fibrotic activity of the screened peptides, the expression levels of three fibrotic indicators (α-SMA, COL1A1 and TGF-β1) were analysed at both the transcriptional and translational levels. HSC-T6 and NIH-3T3 cells were pre-incubated with different concentrations (10–100 μM) of peptides for 1 h and then treated with rat TGF-β1 (5 ng/mL) for 24 h. The data from real-time qPCR (Table 5) showed that the expression levels of these three fibrotic genes were all significantly up-regulated by rat TGF-β1, but the up-regulated mRNA levels could be reduced by the screened peptides. Table 5 shows that Pep70 reduced the level of α-SMA mRNA in a dose-dependent manner in HSC-T6 cells, indicating its potential inhibitory effects on myofibroblast differentiation. Furthermore, a similar suppressive effect of Pep70 against NIH-3T3 cells was also observed. In addition, this peptide caused a dose-dependent attenuation of COL1A1 mRNA levels. The proportional decline in COL1A1 mRNA levels reached 47.6% and 42.8% with a Pep70 dose of 10 μM, confirming that Pep70 abrogated TGF-β-induced collagen protein accumulation. Similarly, the TGF-β1 mRNA level was also down-regulated by Pep70, and this inhibition was more pronounced than for ADP355 at a dose of 100 μM. Interestingly, as shown in Figure 4, among these three screened peptides, Pep70 exhibited the most marked suppression of the protein expression of α-SMA, COL1A1 and TGF-β1 in both *in vitro* cell lines. This inhibitory effect was similar to the positive control peptide ADP355. In NIH-3T3 cells, the average grey value of these three proteins in the Pep70-treated group was much lower than in the model group (2.8 versus 0.9, 2.1 versus 1.1 and 3.4 versus 2.1). Moreover, the decreased intensities of the different protein bands were also observed compared with the model group in TGF-β1-stimulated HSC-T6 cells (2.5 versus 1.2 for α-SMA, 2.4 versus 1.8 for COL1A1 and 3.6 versus 0.9 for TGF-β1). Taken together, Pep70 is believed to be a promising anti-fibrotic peptide.

**Table 5.** The relative mRNA levels of α-SMA, COL1A1 and TGF-β1 in HSC-T6 and NIH-3T3 cells.

		HSC-T6 cells			NIH-3T3 cells		
	Drug (μM)	α-SMA	COL1A1	TGF-β1	α-SMA	COL1A1	TGF-β1
Control	–	1.00 ± 0.15	1.00 ± 0.57	1.00 ± 0.08	1.00 ± 0.04	1.00 ± 0.11	1.00 ± 0.09
Model	–	4.52 ± 0.78	3.33 ± 0.64	1.14 ± 0.11	4.04 ± 0.26	3.40 ± 0.37	1.39 ± 0.17
Pep 69	10	2.60 ± 0.15*	2.04 ± 0.49*	0.65 ± 0.12*	3.51 ± 0.12	1.58 ± 0.50*	0.90 ± 0.12
	50	2.09 ± 0.40**	1.63 ± 0.15*	0.57 ± 0.02*	2.92 ± 0.21	1.32 ± 0.10**	0.58 ± 0.07**
	100	1.93 ± 0.22**	1.01 ± 0.31**	0.40 ± 0.04*	2.54 ± 0.09*	0.93 ± 0.05**	0.68 ± 0.21*
Pep 70	10	2.24 ± 0.37**	1.90 ± 0.10*	0.34 ± 0.04**	2.29 ± 0.11*	1.78 ± 0.31*	0.90 ± 0.06
	50	1.93 ± 0.38**	1.21 ± 0.08**	0.29 ± 0.19**	1.49 ± 0.24**	1.05 ± 0.19**	0.62 ± 0.12**
	100	0.99 ± 0.23**	0.58 ± 0.14**	0.17 ± 0.13**	0.82 ± 0.06**	0.76 ± 0.14**	0.38 ± 0.06**
Pep 71	10	2.98 ± 0.87	2.36 ± 0.08	0.82 ± 0.18	3.81 ± 0.58	3.46 ± 0.57	1.28 ± 0.00
	50	2.95 ± 0.43	1.98 ± 0.16*	0.43 ± 0.05*	2.72 ± 0.09	2.03 ± 0.32	0.89 ± 0.20
	100	2.42 ± 0.58*	1.51 ± 0.21*	0.28 ± 0.03**	2.22 ± 0.49*	1.77 ± 0.75*	0.81 ± 0.23
ADP 355	10	2.01 ± 0.07**	1.85 ± 0.13*	0.42 ± 0.19*	0.99 ± 0.11**	0.57 ± 0.02**	0.43 ± 0.02**
	50	1.08 ± 0.10**	1.03 ± 0.35**	0.38 ± 0.09**	0.57 ± 0.10**	0.54 ± 0.03**	0.41 ± 0.14**
	100	0.98 ± 0.20**	0.80 ± 0.28**	0.25 ± 0.10**	0.42 ± 0.05**	0.44 ± 0.05**	0.38 ± 0.03**
Pep 56	10	4.26 ± 0.65	3.21 ± 0.29	0.93 ± 0.07	3.95 ± 0.44	3.11 ± 0.50	1.27 ± 0.06
	50	3.92 ± 0.59	3.16 ± 0.35	1.03 ± 0.11	4.07 ± 0.58	3.07 ± 0.02	1.17 ± 0.01
	100	3.72 ± 0.47	3.08 ± 0.03	1.11 ± 0.06	3.98 ± 0.52	2.83 ± 0.07	1.14 ± 0.17

* *p* < 0.01.

** *p* < 0.001 versus the model group (only rat TGF-β1 stimulation). These data show the mean of two experiments, and three parallel samples were prepared for each peptide concentration.

### Docking studies

**Figure 5.**
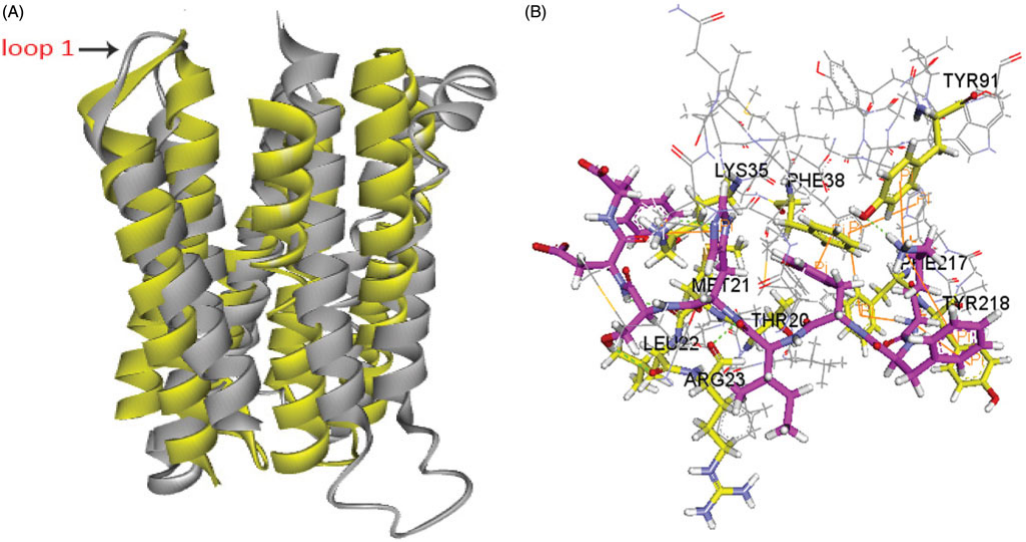
(A) Superposition of our homology model of adiponectin receptor 1 (yellow) and the crystal structure of adiponectin receptor 1 (PDB id: 3WXV; grey; 135–355 region). The RMSD between Cα atoms was 6.64 Å. (B) The binding mode of Pep70 docked to the 3WXV. 3WXV is shown as a line representation. Pep70 (purple) and the residues involved in the interaction (yellow) are displayed as a stick representation. Hydrogen bonds are depicted by the green dotted lines. Pi–pi interactions are depicted by the orange solid lines.

**Figure 6.**
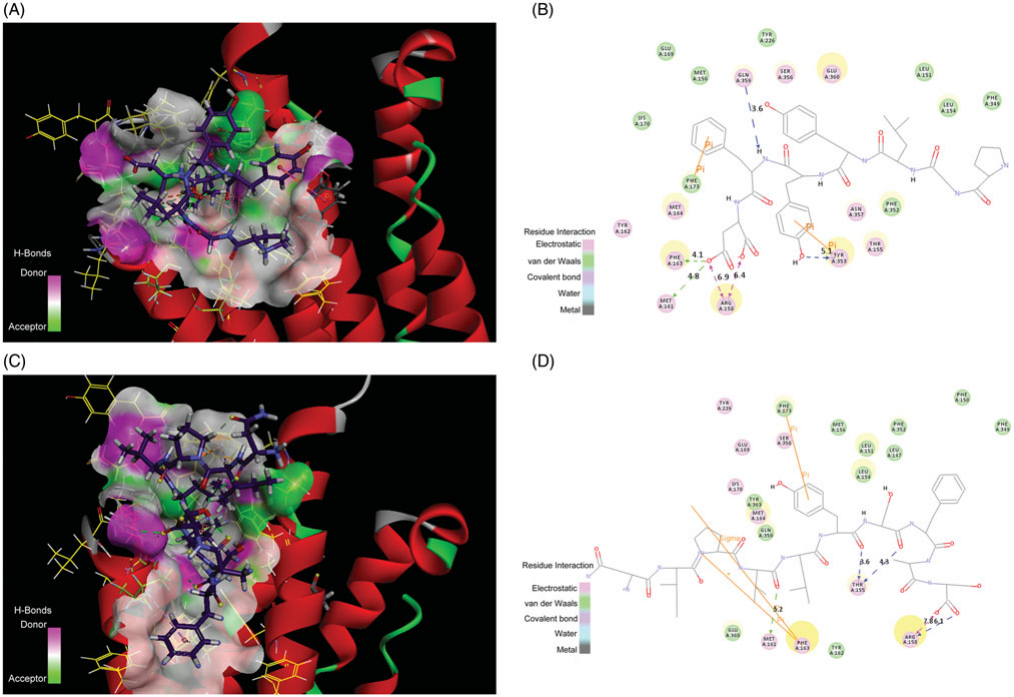
(A) The H-bond interactions between Pep70 and crystal structure 3WVX with the crystal structure of 3WVX shown as a line representation. Pep70 (purple) and the residues involved in the interaction (yellow) are displayed as a stick representation. Hydrogen bonds are depicted by the green dotted lines. Pi–pi interactions are depicted by the orange solid lines. (B) Plot of the interactions between Pep70 and 3WVX. (C) The binding mode of ADP355 to 3WVX. (D) Plot of the interactions between ADP355 and 3WVX.

Interestingly, a crystal structure of human adiponectin receptor 1 was recently published (PBD id: 3WXV), and its structure is similar with our homology model, especially the loop1 (Figure 5A). Meanwhile, docking the peptides to the crystal structure led to a similar result with our model (Figure 5B) in that Lys35 (Lys170 in 3WVX) was in both interactions. Additionally, the docking pose analysis of Pep70 (Figure 6) revealed hydrogen bonding interactions and π–π interactions that exist in both peptide–protein complexes. Combined with the H-bonding surface plot, the binding site of the complex was shown as a long and narrow cavity. Pep70 was led to entering the cavity through the hydrogen bonding interactions with Gln359, Arg158 and Tyr353. In addition, π–π interactions were formed with Phe173 and Tyr353, which allowed the entire Pep70 peptide to be embedded in the cavity. Compared with the docking pose of ADP355 (Figure 6), the most active AdipoR1 agonist peptide Pep70 shared a similar binding mode, including a self π–π interaction with Phe173 of 3WVX and H-bond interactions with Arg158 of 3WVX that stabilised their structures. In addition, for Pep70, a π–π interaction formed with Phe173 of 3WVX makes Pep70 embed deeper into the cavity, which may potentially make Pep70 show a comparable activity to ADP355.

## Conclusions

In conclusion, we constructed the structure of AdipoR1 by homology modelling *in silico*. The putative active site of the model was further explored by docking to known active peptides. Virtual screening was carried out with a set of 747 manually designed peptides. Three peptides with high docking scores were evaluated *in vitro* for their anti-fibrotic activities, and the novel peptide Pep70 with promising anti-fibrotic effects was obtained. However, to achieve a higher level of bioactivity, further studies of Pep70 are needed.

The work presented here provides a reliable model of AdipoR1 and a better understanding of the interactions between the peptide and AdipoR1. Virtual screening and biological activity studies on early leads provided initial validation of this approach and will facilitate screening for additional AdipoR1 agonists.
